# Supplementary material for: Insights into the Fe3+ Doping Effects on the Structure and Electron Distribution of Cr2O3 Nanoparticles
Source: Nanomaterials (Basel). 2023 Mar 8;13(6):980. doi: 10.3390/nano13060980 (PMC10059910; doi:10.3390/nano13060980)
Supplement: Supplementary file 1 [file nanomaterials-13-00980-s001.zip › nanomaterials-2220042-supplementary.pdf]

Insights into the Fe^{3+}-doping effects on the structure and electron distribution of Cr_2O_3 nanoparticles

Cledson Santos[1,2], John M. Attah-Baah[1,2], Romualdo S. Silva Junior[1,2], Marcelo A. Mâcedo[1,2], Marcos V. S. Rezende[1], Robert S. Matos[3], Ştefan Ţălu[4], Dung Nguyen Trong[5, †], Simone P. A. da Paz[6], Rômulo S. Angélica[6], Nilson S. Ferreira[1,2,7,*]

[1] Department of Physics, Federal University of Sergipe, 49100-000, São Cristóvão, SE, Brazil

[2] Laboratory of Corrosion and Nanotechnology (LCNT), Federal University of Sergipe, 49100-000, São Cristóvão, SE, Brazil

[3] Amazonian Materials Group, Federal University of Amapá, 68902-280, Macapá, AP, Brazil

[4] The Directorate of Research, Development and Innovation Management (DMCDI), Technical University of Cluj-Napoca, 15 Constantin Daicoviciu St., 400020 Cluj-Napoca, Romania

[5] Faculty of Physics, Hanoi National University of Education, 136 Xuan Thuy, Cau Giay, Hanoi, 100000, Vietnam

[6] Institute of Geosciences, Federal University of Pará, 66075-110, Belém, PA, Brazil

[7] PPGCA, Universidade Federal do Amapá, 68902-280, Macapá, AP, Brazil

† Correspondence: dungntdt2018@gmail.com (D.N.T.)

* Correspondence: nilson@academico.ufs.br (N.S.F.)

Table S1: Crystal data and refined parameters for $Cr_{2-x}Fe_xO_3$ ($0.0 \leq x \leq 0.18$) nanoparticles obtained from X-ray diffraction.

**Parameters**	$Cr_{2-x}Fe_xO_3$			
	**x = 0.0**	**x = 0.06**	**x = 0.12**	**x = 0.18**
**Crystal system**	Rhombohedral	Rhombohedral	Rhombohedral	Rhombohedral
**Space group**	R -3c (167)	R -3c (167)	R -3c (167)	R -3c (167)
**Lattice parameters**				
a = b (Å)	4.957243	4.957635	4.959920	4.960923
c (Å)	13.594714	13.595851	13.599133	13.598163
c/a	2.742394	2.742407	2.741805	2.741055
$\alpha = \beta$ (°)	90.00	90.00	90.00	90.00
γ(°)	120.0	120.00	120.00	120.00
Unit cell volume ($Å^3$)	289.322	289.392	289.729	289.825
X-ray density (g/cm^3)	5.245	5.253	5.261	5.269
**Atomic coordinates**				
Cr^{3+}/Fe^{3+}				
x	0	0	0	0
y	0	0	0	0
z	0.34765	0.34702	0.34783	0.34780
Biso	1.82691	2.04750	2.24590	1.81561
Occupancy	0.35246/0	0.33757/0.02426	0.32969/0.03638	0.31478/0.04148
Wyckoff notations	12c	12c	12c	12c
O^{2-}				
x	0.31465	0.31061	0.30636	0.30669
y	0.00000	0.00000	0.00000	0.00000
z	1/4	1/4	1/4	1/4
Biso	0.0	0.0	0.0	0.0
Occupancy	0.51913	0.51427	0.51639	0.51148
Wyckoff notations	18e	18e	18e	18e
**Microstructure**				
Average crystallite size (nm)	22.9 ± 2.8	33.5 ± 4.3	43.2 ± 3.0	75.5 ± 7.7
Crystallite Apparent size 010 (nm)	22.9	31.0	43.5	77.3
Crystallite Apparent size 001 (nm)	25.1	43.5	47.4	87.5
Crystallite Apparent size 100 (nm)	18.1	29.2	41.4	68.2
Microstrain $\langle \varepsilon \rangle \times 10^{-4}$	11.2 ± 2.5	5.2 ± 1.7	5.8 ± 1.4	1.4 ± 0.8
**Bond Valence Sum**				
Cr^{3+}	2.903	3.014	3.102	3.345
Fe^{3+}	-	3.609	3.519	3.126
O^{2-}	1.935	1.992	2.084	1.972
**Refinement parameters**				
R_P	6.20	6.14	5.59	5.40
R_{WP}	7.90	7.83	7.15	6.82
R_{Exp}	6.82	6.98	6.62	6.19
χ^2	1.34	1.26	1.16	1.21
R_d (%)	100	99.59	98.89	94.85

**Bond lengths (Å)**				
(Cr/Fe)-O1	2.047(9)	2.033(7)	2.024(6)	2.036(7)
(Cr/Fe)-O2	1.943(2)	1.952(2)	1.958(3)	1.945(8)
<Cr/Fe-O>	1.995(6)	1.992(9)	1.991(5)	1.991(3)
**Angles (degree)**				
(Cr/Fe)-O-(Cr/Fe)	80.3(4)	80.8(3)	82.5(4)	82.9(1)
